# Effect of *Erythrina variegata* seed extract on hyperlipidemia elicited by high-fat diet in wistar rats

**DOI:** 10.4103/0975-7406.72139

**Published:** 2010

**Authors:** G. Balamurugan, A. Shantha

**Affiliations:** Department of Pharmacology, C. L. Baid Metha College of Pharmacy, Jyothi Nagar, Thoraipakkam, Chennai - 600 097,Tamil Nadu, India; 1 Department of Pharmaceutical Analysis, C. L. Baid Metha College of Pharmacy, Jyothi Nagar, Thoraipakkam, Chennai - 600 097, Tamil Nadu, India

**Keywords:** Antioxidant enzymes, *Erythrina variegata*, high fat diet, HMG-CoA reductase, lipid profile, β-sitosterol

## Abstract

**Objective:**

To investigate the effect of the methanolic extract of *Erythrina variegata* (Linn.) var Orientalis (Fabaceae) seeds (MEEV) in reducing the cholesterol levels and as well as antioxidant in experimentally induced hyperlipidemic rats.

**Materials and Methods:**

Doses of 200 and 400 mg/kg of the extract were evaluated for its effect on lipid profile, HMG-CoA reductase, and on antioxidant enzymes in high-fat diet (HFD) induced hyperlipidemia.

**Results and Conclusion:**

The elevated levels of total cholesterol, triglycerides, low-density lipoprotein, and very low density lipoprotein due to HFD was reduced by concurrent treatment with MEEV (200 and 400 mg/kg) significantly (*P*<0.001). A significant reduction (*P*<0.001) in high-density lipoprotein was noticed in HFD fed groups; however, a nonsignificant increment was produced by the administration of MEEV (200 and 400 mg/kg). The HMG-CoA reductase activity was increased in HFD fed animals significantly (*P*<0.001) and was reduced by MEEV 400 mg/kg significantly (*P*<0.001). There was a noticed increase in the body weight and mesenteric fat pad weight in HFD fed group (*P*<0.001), which was reduced by the administration of MEEV (200 and 400 mg/kg). The antioxidant enzymes such as superoxide dismutase and catalase were reduced significantly in the HFD fed group, whose levels were increased significantly (*P*<0.001) by the administration of MEEV (200 and 400 mg/kg). Lipid peroxidation was increased in HFD fed animals, which was reduced significantly (*P*<0.001) by the treatment with MEEV (200 and 400 mg/kg).

Hyperlipidemia has been ranked as one of the greatest risk factors contributing to the prevalence and severity of coronary heart disease.[1] Coronary heart disease, stroke, atherosclerosis, and hyperlipidemia are the primary cause of death in developed countries.[2] Hyperlipidemia is characterized by elevated serum total cholesterol, low-density (LDL) and very low density lipoprotein cholesterol (VLDL) with decreased high-density lipoprotein (HDL) levels. Hyperlipidemia-associated lipid disorders are considered to cause atherosclerotic cardiovascular disease.[3] Among these hypercholesterolemia and hypertriglyceridemia are closely related to ischemic heart disease.[4] The main aim of the treatment in patients with hyperlipidemia is to reduce the risk of developing ischemic heart disease or the occurrence of further cardiovascular or cerebrovascular disease.[5] Currently available hypolipidemic drugs have been associated with a number of side effects.[6] The consumption of synthetic drugs leads to hyperuricemia, diarrhea, nausea, myositis, gastric irritation, flushing, dry skin and abnormal liver function. More than thirteen thousands plants have been studied for various pharmacological properties.[7] Hyperlipidemia is classified into a primary and a secondary type, which indicates the complexities associated with the disease. The primary disease may be treated by antilipidemic drugs but the secondary type originating from diabetes, renal lipid nephrosis, or hypothyroidism demands the treatment of the original disease rather than hyperlipidemia.[8] High-fat diet (HFD) may lead to the production of extra VLDL, resulting in the formation of large amounts of LDL, which may stick to the walls of the blood vessels if the quantity of HDL is insufficient, resulting in blockades for the normal flow of blood. Therefore improvement in human diet is highly recommended for disease prevention.[9]

*Erythrina variegata* Linn. var *Orientalis* (Linn.) Merrill (Family: Fabaceae) is a medium sized tree with smooth yellowish or grayish, shinning bark cultivated in many parts of India especially in Southern India often for ornamental purpose. The flowers are coral red and serve the ornamental purpose.[10] The whole plant including the seeds is used for a variety of illness in Indian system of medicine. The leaves were used to relieve pain and inflammation when crushed and applied to rheumatic joint. Fresh juice of the whole plant is used to cure long-standing dysmenorrhea and remove sterility in fatty women by gradually reducing abdominal fat and producing natural menstrual flow.[11] In Siddha system, the entire plant and seeds are used for the treatment of stomatitis, dysentery, sterility, diabetes, and eye disorders, and different parts of the plant are also used as sedative, antiepileptic, and as an antiseptic.[10] The entire plant including the seeds are reported to possess antihypertensive,[12] antimicrobial,[13] sedative,[14] immunosuppressive,[15] and anti-inflammatory properties.[16]

Phytochemical investigations on the plant revealed the presence of alkaloids,[10,17] flavanoids and isoflavanoids,[18–22] phenyl coumarins,[23] lectins,[24] flavones glycosides,[25] steroids, and fatty acids.[26] The seeds of *E. variegata* contain a pale yellow fixed oil which contains unsaturated fatty acid (oleic and linoleic acids) along with β-sitosterol, campesterol, and stigmasterol in the seed coat,[27] a very useful phyto constituent with antilipidemic effects. Hence, the work was carried out to evaluate the hypolipidemic activity of methanolic extract of *E. variegata* seeds (MEEV) on rats fed with HFT.

## Materials and Methods

### Animals

Male Wistar rats weighing 200-250 g obtained from the animal house of C. L. Baid Metha College of Pharmacy were used in the study. The animals were maintained in well ventilated rooms with 12: 12 h light/dark cycle in polypropylene cages. All animals were acclimatized to the laboratory conditions one week prior to the initiation of the study. The study proposal was approved by Institutional animal Ethical Committee (IAEC) constituted under CPSCEA [Approval No: IAEC/XIII/02/CLBMCP-2009-2010 dated 22-03-09].

### Plant material and extraction

The seeds of *E. variegata* were collected from the villages around Chennai, Tamil Nadu, India. The identity of the seeds was confirmed by Dr. Sasikala Ethirajulu, Ph. D, Research Officer, Central Research Institute of Siddha, Chennai, Govt. of India. A voucher specimen (CLMBCP-III- EV-17/08) was deposited in the department of Pharmacology, C. L. Baid Metha College of Pharmacy, Chennai. The seeds were further shade-dried for 72 h and powered with an electric grinder. The coarse powder was extracted with methanol and the residue was evaporated under reduced pressure in rotary vacuum evaporator at 60°C to obtain a dark brown colored molten mass. The percentage yield was found to be 12.7% w/w.

### Chemicals and drugs

Standard kits used for the estimation of total cholesterol, triglyceride, and HDL-cholesterol were purchased from Keragen Technologies, Bangalore, India. Total protein kit was purchased from Sigma, USA. Other chemicals and solvents were purchased from s.d. Fine chemicals, Mumbai, India.

### Acute toxicity studies

Wistar rats weighing 200–250 g (three nos) were used in the procedure. Acute oral toxicity was performed as OECD-423 guidelines.[28] The animals were fasted overnight, provided only water after which extract was administered to the animals orally at the dose level of 5 mg/kg body weight by gastric intubation and the animals were observed for 24 h. If mortality was observed in two or three animals, then the dose administered was assigned as a toxic dose. If mortality was observed in one animal, then the same dose was repeated again to confirm the toxic dose. If mortality was not observed, the procedure was repeated for further higher doses such as 50, 300 and 2000 mg/kg body weight. The animals were observed for toxic symptoms such as behavioral changes, locomotion, convulsions, and mortality for further 72 h.

### Induction of hyperlipidemia

#### Preparation of HFT

Semisynthetic basal diet for the rats was prepared which contains (g/100 g) casein 30 g; agar 2 g; cellulose-8 g; sucrose-51 g; vitamin mixture-0.58 g and mineral mixture-0.50 g.[29] Hyperlipidemia was induced by addition of cholesterol-1.5 g and coconut oil 8 ml for every 100 g of basal diet.[30]

### Experimental design

The animals were divided into five groups containing six animals each. Group I served as control; group II received HFD; group III received HFD and MEEV 200 mg/kg; group IV HFD and MEEV 400 mg/kg and group V received HFD and atorvastatin 1.2 mg/kg for 90 days.[31] Replenishing a known quantity of fresh food daily at 10.30 a.m. and thereby measuring the food intake of the previous day carried out measurement of daily food consumption. Body weight of rats was recorded weekly to assess percentage of weight gain in each group. General well-being and behavior of the animals were observed daily throughout the period of study. The litter in the cage was renewed twice a week to ensure maximum comfort for the animals. Animals were kept starved overnight on the 90th day. On the next day all the animals were sacrificed under light ether anesthesia. Blood was collected by carotid bleeding into sterilized dry centrifuge tubes and allowed to coagulate for 30 min at 37°C. The clear serum was separated at 2500 rpm for 10 min and was kept in frozen containers.

### Estimation of cholesterol and lipids

The separated serum was subjected to biochemical estimation of parameters such as total cholesterol,[32] triglycerides,[33] and HDL-cholesterol.[32,34] LDL-cholesterol, VLDL-cholesterol, and the atherogenic index were calculated using standard equations.[35]

$$\mathrm{VLDL}-\mathrm{cholesterol}\;=\;\frac{\mathrm{Towtal}\;\mathrm{serum}\;\mathrm{triglyceride}}{5}$$

$$\mathrm{LDL}-\mathrm{cholesterol}\;=\;\frac{\mathrm{total}\;\mathrm{serum}\;\mathrm{cholesterol}\;-\mathrm{total}\;\mathrm{serum}\;\mathrm{triglycerides}\;-\;\mathrm{HDL-C}}{5}$$

$$\mathrm{Atherogenic}\;\mathrm{index}\;=\;\frac{\mathrm{total}\;\mathrm{serum}\;\mathrm{cholesterol}}{\mathrm{total}\;\mathrm{serum}\;\mathrm{HDL-C}}$$

The percentage of lipid-lowering effect was calculated according to the following equation[36]:

$$\%\;\mathrm{lipid}\;\mathrm{lowering}\;=\;\frac{\mathrm{HFD-fed}\;\mathrm{animals}\;\mathrm{lipid}\;\mathrm{values}\;-\;\mathrm{drug}\;+\;\mathrm{HFD-fed}\;\mathrm{animals}\;\mathrm{lipid}\;\mathrm{value}\;\times \;100}{\mathrm{HFD-fed}\;\mathrm{animals}\;\mathrm{lipid}\;\mathrm{value}}$$

### Estimation of HMG-CoA reductase

The liver tissue was removed as quickly as possible and a 10% homogenate was prepared in saline arsenate solution. The homogenate was deproteinized using an equal volume of dilute perchloric acid and allowed to stand for 5 min followed by centrifugation; 0.5 ml of freshly prepared hydroxylamine reagent was added to 1 ml of the filterate. It was mixed and 1.5 ml of ferric chloride reagent was added after 5 min. The absorbance was read after 10 min at 540 nm against a similarly treated saline arsenate blank. The ratio of HMG-CoA/mevalonate was calculated.[37]

### Determination fat pad weight

After exsanguination of animals, mesenteric fat pads were removed and weighed.[38]

### Estimation of antioxidant enzymes

Hundred milligrams of liver tissue was weighed and homogenate was prepared in 10 ml Tris hydrochloric acid buffer (0.5 M; pH 7.4) at 4°C. The homogenate was centrifuged and the supernatant was used for the assay of antioxidant enzymes namely catalase,[39] superoxide dismutase,[40] and lipid peroxidation (LPO).[41]

### Statistical analysis

One-way analysis of variance (ANOVA) followed by Dunnet’s *t*-test was applied to determine the statistical differences among experimental groups. The level of significance was set at 0.05.

## Results and Discussion

MEEV did not produce any toxic symptom or mortality up to the dose level of 2000 mg/kg body weight orally in mice, and hence the drugs were considered safer for further pharmacological screening. According to OECD-423 guidelines for acute oral toxicity, the LD _50_ dose of 2000 mg/kg and above is categorized as unclassified.

There was a significant increase (*P*<0.001) in the level of total cholesterol, triglycerides, low–density, and very low density lipoprotein in the group II animals fed with HFT in comparison with the normal untreated control animals. Treatment with MEEV at 200 and 400 mg/kg produced a reduction of (24.15% and 32.32%) in total cholesterol; (14.43% and 19.24%) in triglycerides; (28.7% and 38.9%) in LDL in the respective doses with a statistical significance of (*P*<0.001) in comparison with group II animals. There was a moderate reduction in the VLDL in the group III animals (12.92%) and a significant reduction in the group IV animals (19.21%) in comparison with the group II animals. The reduction in the HDL produced by the group of animals fed with HFT was significant (*P*<0.001) in comparison with group I animals. However, the treatment with MEEV 200 and 400 mg/kg increased the HDL level by 17.73% and 20.13% in the respective groups but was nonsignificant statistically. The restoration of all the altered lipid parameters by the group V animals treated with atorvastatin 1.2 mg/kg was statistically significant (*P*<0.001) and comparable with normal animals. There was a significant (*P*<0.001) increase in the HMG-CoA reductase activity in the group II animals indicating increased cholesterol synthesis. The elevated enzyme activity was reduced significantly by treatment with MEEV (200 and 400 mg/kg) dose dependently (*P*<0.01 and 0.001, respectively) and so by the standard drug atorvastatin 1.2 mg/kg. The atherogenic index (AI) of the animals fed on HFT was increased many fold in group II animals in comparison with normal group I animals (*P*<0.001), whereas treatment with MEEV reduced the index dose dependently with a statistical significance (*P*<0.001) in comparison with group II animals [Table 1].

**Table 1:** Effect of MEEV on lipid parameters, body weight, and fat pad weight in normal and hypercholesterolemic rats

Parameters	Group 1	Group 2	Group 3	Group 4	Group 5
TC (mg/dl)	79.25±0.42	203.80±0.49^a^***	162.16±0.45^b^***	143.61±0.49^b^***	73.30±0.33^b^***
TG (mg/dl)	58.9±0.74	135.1±0.37^a^***	115.6±0.42^b^***	109.1±0.51^b^***	55.3±0.19^b^***
HDL-c (mg/dl)	29.32±0.63	15.00±0.41^a^***	16.16±0.80bNS	16.82±0.28bNS	17.02±0.21^b^***
LDL-c (mg/dl)	73.34±0.48	179.78±0.97^a^***	142.48±0.48^b^***	125.19±0.41^b^***	65.64±0.63^b^***
VLDL-c (mg/dl)	11.78±0.54	27.01±0.65^a^***	23.52±0.88^b^*	21.82±1.02^b^***	11.06±1.03^b^***
Atherogenic index	2.70±0.54	13.58±0.42^a^***	10.03±0.64^b^***	8.53±0.56^b^***	4.30±0.74^b^***
HMG-CoA/Mevalonate ratio	11.24±0.83	3.31±0.12^a^***	5.86±0.34^b^**	7.32±0.49^b^***	7.89±0.37^b^***
Body weight (g)	249.67±4.12	327.57±5.89^a^***	284.24±4.43^b^***	269.16±2.72^b^***	233.84±3.44^b^***
Mesenteric fat pad weight (g)	0.28±0.12	0.87±0.29^a^**	0.40±0.03^b^*	0.31±0.18^b^**	0.17±0.08^b^***

Values are mean ± SEM, *n*=6 animals in each group. Comparisons were made between: a, group I vs. II and b, group II vs. III, IV, and V. Symbols represent statistical significance:

* *P*<0.05,

** *P*<0.01,

*** *P*<0.001

The body weight of group II animals were increased significantly (*P*<0.001) in comparison with normal control group I animals. The increment in the weight was reduced significantly (*P*<0.001) by the administration of MEEV (200 and 400 mg/kg) as well as atorvastatin 1.2 mg/kg in comparison with the HFD fed rats. The mean increase in the mesenteric fat pad was significant (*P*<0.01) in animals fed with HFD due to accumulation of cholesteryl esters. Treatment with MEEV (200 and 400 mg/kg) reduced the elevated fat pad weight significantly (*P*<0.05 and <0.01, respectively). However, treatment group receiving atorvastatin 1.2 m/kg produced maximum reduction in fat pad weight (*P*<0.001) [Table 1].

The levels of antioxidant enzymes such as SOD and CAT were reduced significantly (*P*<0.001) in animals treated with HFD. The administration of MEEV at doses 200 and 400 mg/kg significantly restored the reduced enzymic activity (*P*<0.001). Feeding with HFD produced an increased LPO in the group II animals (*P*<0.001), which was significantly reduced by the treatment with MEEV (200 and 400 mg/kg) in group III and IV animals, respectively [Table 2].

**Table 2:** Effect of MEEV on antioxidant enzymes in normal and hypercholesterolemic rats

Parameters	Group 1	Group 2	Group 3	Group 4	Group 5
SOD (Units/mg of protein)	6.90±0.10	3.13±0.12^a^***	4.36±0.16^b^**	4.69±0.12^b^***	5.65±0.17^b^***
CAT (n moles of H_2_O_2_ decomposed/mg of protein)	44.60±1.32	22.24±1.25^a^***	31.18±1.47^b^***	36.75±1.31^b^***	39.28±1.33^b^***
LPO (n moles of TBRAS/mg of protein)	2.72±0.04	4.40±0.05^a^***	3.80±0.06^b^***	3.20±0.06^b^***	2.90±0.06^b^***

Values are mean ± SEM, *n*=6 animals in each group. Comparisons were made between: a, group I vs. II and b, group II vs. III, IV, and V. Symbols represent statistical significance:

* *P*<0.05,

** *P*<0.01,

*** *P*<0.001

It is well known that increased HDL-cholesterol levels have a protective role in coronary artery disease.[42] Similarly increased level of serum LDL-cholesterol results in increased risk for the development of atherosclerosis.[43] The increased level of HDL-cholesterol and decreased cholesterol along with its LDL fraction, which is evident from the results, could be due to increased cholesterol absorption through gastrointestinal tract. Thus the decreasing cholesterol levels in the body under the influence of *E. variegata* could have been due to rapid catabolism of LDL-cholesterol through its hepatic receptors for final elimination in the form of bile acids.[44]

As to the data of HDL, there was a significant increase in the serum HDL level in the rats treated with MEEV and also play a key role in the protection against oxidative damage of membrane.[45,46] Therefore, the atherogenic index (ratio of TC to HDL) is an important prognostic marker for cardiovascular disease.[47–49] Lower ratio of TC to HDL is usually associated with a low HDL and/or elevated TC.

The presence of β-sitosterol has also been reported to be a useful phytoconstituent in the treatment of hyperlipidemia.[50] The seed coat of *E. variegata* has already been reported to contain β-sitosterol along with stigmasterol and campesterol. β-Sitosterol is a plant sterol, a compound similar to cholesterol. It is not absorbed much from the digestive system to the rest of the body, so it works mostly within the digestive system. It works by blocking the absorption of cholesterol from the intestine. Because β-sitosterol decreases the absorption of cholesterol in the intestine, it can decrease cholesterol levels in the body. Stigmasterol and campesterol have effects similar to β-sitosterol, but campesterol produces only a modest lipid-lowering effect on par with β-sitosterol.[51]

The increased HMG CoA/Mevalonate ratio noted in the extract-treated group in comparison with HFD-treated group could be due to an increased cholesterol excretion and/or decreased cholesterol absorption. The HMG-CoA/mevalonate has an inverse relationship to the activity of HMG-CoA reductase. HMG-CoA reductase catalyzes an early, rate-limiting step in cholesterol biosynthesis. HMG-CoA reductase inhibitors (statin group of drugs) like atorvastatin, simvastain, and rosuvastatin reduces raised triglyceride levels caused by elevated VLDL levels. By reducing the conversion of HMG-CoA to mevalonate, the statins inhibit hepatic cholesterol biosynthesis.[52] The results of our study indicate a similar kind of reduction in LDL, VLDL, and triglycerides as like the group treated with atorvastatin 10 mg/kg. Statins reduce the LDL-cholesterol levels by enhancing the removal of its precursors (such as VLDL and IDL) and also by decreasing hepatic VLDL production.[52] In addition, the statins tend to increase the synthesis of LDL-receptors and their degradation may be reduced upon its administration. This mechanism likely account for the triglyceride lowering effect of statins and a similar effect is noticed in *E. variegata* seed extract treated groups and may be the likely mechanism of action for reduced LDL and triglyceride level.

Hypercholesterolemia along with high-cholesterol diet and oxidative stress increases serum LDL levels resulting in increased risk for the development of atherosclerosis.[43] Endothelial cells bind LDL and when activated by injury, these cells are attached to monocytes/macrophages and generate free radicals, which oxidize LDL, resulting in LPO and destruction of the receptor needed for the normal receptor-mediated clearance of LDL.[53] Atorvastatin is well known to prevent LPO and increases the LDL receptor expression.[52] In addition, atorvastatin has additional antioxidant property.[54] From the results, it becomes evident that treatment with MEEV also reduced the LPO in a dose-dependent manner, which may be through the mechanism similar to that of statin.

The antioxidant enzymes mainly SOD and CAT are known to control oxidative damage.[55] Catalase is a common enzyme whose functions include catalyzing the decomposition of H_2_O_2_ to H_2_O and O_2_. Catalase has the highest turnover rates of all enzymes; one molecule can convert millions of molecules of hydrogen peroxide to water and oxygen per second. Similarly Superoxide dismutase (SOD) is an enzyme that removes the superoxide (O2^-^) radical, repair cells, and reduces the damage done to them by superoxide.[56] The present study indicates that the animal groups treated with *E. variegata* seed extract had higher levels of antioxidative enzymes (CAT and SOD) and decreased level of LPO indicating its efficacy to reduce the LDL-cholesterol oxidation and atherogenesis. Phytoconstituents such as flavonoids and poly phenols are natural antioxidants that may reduce oxidative process and increase SOD, CAT activities.[57,58] The similar phytoconstituents are reported in E. variegate, which adds on to their efficacy in increased SOD, CAT activities significantly.[18,19] It was reported that these compounds act mainly as promoters for SOD, CAT.[59] The currently noted elevated levels of both CAT and SOD with MEEV treatment could be due to the presence and influence of flavonoids.[55] Reduced level of SOD and CAT observed in HFD-treated group, therefore, causes a number of deleterious effects to cells and tissues due to the accumulation of potent superoxide radical and hydrogen peroxide.

Administration of HFD produced a highly significant increase in weight mesenteric fat pads. A reduction in the raised weight in the fat pads as observed in the groups of animals treated with MEEV may be attributed to increased thermogenesis and decreased lipogenesis.[38]

Furthermore, there was also an increase in cholesterol content of the fecal matter indicating that *E. variegata* promoted the excretion of cholesterol (data not shown). Bioactive fractions of plant origin are often known to reduce dyslipidemia by this mechanism too.[60]

In conclusion, from the observation, the protective effect of the seeds of *E. variegata* on high fat induced hyperlipidemia may be attributed to a decrease in cholesterol synthesis, an increase in cholesterol excretion and expression of LDL receptor and subsequent catabolism. The antioxidant effect afforded may be playing a promising role in retarding/preventing associated cardiovascular complications secondary to hyperlipidemia. Studies on the isolated fractions and constituents are needed to elucidate mechanisms by which *E. variegata* exert protective effects on hyperlipidemia.
